# Relationship between the Sequencing and Timing of Vocal Motor Elements in Birdsong

**DOI:** 10.1371/journal.pone.0143203

**Published:** 2015-12-09

**Authors:** Andrew M. M. Matheson, Jon T. Sakata

**Affiliations:** Department of Biology, McGill University, Montreal, Quebec, Canada; Texas Christian University, UNITED STATES

## Abstract

Accurate coordination of the sequencing and timing of motor gestures is important for the performance of complex and evolutionarily relevant behaviors. However, the degree to which motor sequencing and timing are related remains largely unknown. Birdsong is a communicative behavior that consists of discrete vocal motor elements (‘syllables’) that are sequenced and timed in a precise manner. To reveal the relationship between syllable sequencing and timing, we analyzed how variation in the probability of syllable transitions at branch points, nodes in song with variable sequencing across renditions, correlated with variation in the duration of silent gaps between syllable transitions (‘gap durations’) for adult Bengalese finch song. We observed a significant negative relationship between transition probability and gap duration: more prevalent transitions were produced with shorter gap durations. We then assessed the degree to which long-term age-dependent changes and acute context-dependent changes to syllable sequencing and timing followed this inverse relationship. Age- but not context-dependent changes to syllable sequencing and timing were inversely related. On average, gap durations at branch points decreased with age, and the magnitude of this decrease was greater for transitions that increased in prevalence than for transitions that decreased in prevalence. In contrast, there was no systematic relationship between acute context-dependent changes to syllable sequencing and timing. Gap durations at branch points decreased when birds produced female-directed courtship song compared to when they produced undirected song, and the magnitude of this decrease was not related to the direction and magnitude of changes to transition probabilities. These analyses suggest that neural mechanisms that regulate syllable sequencing could similarly control syllable timing but also highlight mechanisms that can independently regulate syllable sequencing and timing.

## Introduction

Behavior consists of motor gestures that are sequenced and timed in precise ways. The temporal coordination of motor gestures into larger behavioral units is important for the performance of complex behaviors [1–4]. This is particularly true for communicative behaviors in which the sequencing and timing of vocal motor elements could convey different messages [5–8]. While many studies have independently examined the regulation and plasticity of motor sequencing and timing, relatively little is known about the relationship between motor sequencing and timing (e.g., [9–11]).

Birdsong is a vocal communicative behavior composed of distinct acoustic elements (‘syllables’) that are sequenced and timed in a precise manner [5, 8, 12–15]. Adult songbirds use song during social interactions with conspecifics, and the temporal patterning of syllables can change depending on the social circumstances and environment [16–19]. Discrete and specialized neural circuits underlie the moment-by-moment control of birdsong (e.g., [20–25]), and these circuits are analogous to pathways underlying the control of complex human behaviors such as speech, language, and music performance [26–29]. Therefore, analyzing the relationship between the sequencing and timing of vocal motor elements in birdsong can provide insight into general mechanisms of motor control.

While much is known about the independent control of syllable sequencing and syllable timing in birdsong, only a handful of studies have investigated experimental effects on both syllable sequencing and timing (e.g., [16, 30–35]). These studies provide evidence that the control of syllable sequencing and timing could be linked or independent. For example, targeted perturbations of auditory feedback, manipulations of social context, and age affect both syllable sequencing and timing in adult Bengalese finches [16, 19, 30–31, 34], and developmental changes to syllable sequencing correlate with developmental changes in syllable timing in zebra finches [33]. However, many of these studies examined syllable sequencing and timing at distinct parts of song and, thus, only indirectly demonstrate a relationship between the control of syllable sequencing and timing. Indeed, other studies have documented that syllable timing can significantly change without changes to syllable sequencing and vice versa, suggesting the existence of mechanisms that independently regulate syllable sequencing and timing (e.g., [16–18, 34–37]).

Here we directly analyzed the relationship between syllable sequencing and timing in adult Bengalese finch song. Bengalese finches (*Lonchura striata* var. *domestica*) provide an excellent opportunity to assess this relationship because of the relatively complex temporal patterning of syllables in their songs [16, 30, 32, 34–36, 38–39]. In particular, songs of adult Bengalese finches consist of phrases of syllables that vary in their sequencing across renditions (i.e., branch points), and we can analyze how variation in syllable sequencing at branch points relates to variation in the timing of sequence transitions. We first investigated the degree to which the probabilities of transitions within branch points were systematically related to the duration of silent gaps between syllables in the transition. Thereafter, we examined whether naturally occurring changes to syllable sequencing and timing followed this relationship. Specifically, we analyzed the degree to which the magnitudes of long-term age-dependent and acute social context-dependent changes to syllable sequencing correlated with the magnitudes of age- and context-dependent changes to gap durations.

## Materials and Methods

### Animals

Male Bengalese finches were born and raised in a colony at the University of California, San Francisco (n = 10) or purchased from vendors as juveniles and sent to McGill University (2–3 months old; Exotic Wings and Things, Ontario, Canada; n = 12 birds). Birds were socially housed on a 14:10 light-dark photoperiod and provided with food and water *ab libitum*. All procedures were in accordance with the animal care protocols approved by University of California San Francisco Institutional Animal Care and Use Committee (#AN080388) or the McGill University Animal Care and Use Committee (#5977) as well as with guidelines of the Canadian Council on Animal Care.

### Song recording and data collection

Birds were individually housed in sound-attenuating chambers for all song recordings (Acoustic Systems, Austin, Texas; TRA Acoustics, Ontario, Canada). Sound was recorded using an omnidirectional microphone (Countryman Associates, Inc, Menlo Park, CA) positioned above the male’s cage. Sound-activated recording software was used to detect and digitize song (EvTAF (E. Tumer, UCSF), digitized at 32 kHz; Sound Analysis Pro (http://ofer.sci.ccny.cuny.edu/html/sound_analysis.html,v.1.04), digitized at 44.1 kHz). Recorded songs were filtered at 0.3–8 kHz for analysis using software in the Matlab Programming language (The MathWorks, Natick, MA).

Song was recorded from adult Bengalese finches under different social contexts and at different ages to assess the general relationship between syllable sequencing and timing as well as the relationship between acute and long-term changes to syllable sequencing and timing (e.g., [34,35]). To assess context-dependent changes to song, we collected renditions of undirected (UD) and female-directed (FD) song in a subset of young adult Bengalese finches (n = 14 birds; 4–7 months of age; 43.6±5.2 (mean±SEM) songs; range: 21–71 songs). Undirected songs are spontaneous songs produced in isolation, whereas FD songs are produced during courtship interactions with females. To elicit FD song, we briefly (<30 sec) placed a cage with a female adjacent to the male’s cage [16, 19, 31, 40]. Female-directed songs are generally produced within seconds after the introduction of the female and are readily distinguishable from UD songs because they are produced after a male approaches or faces the female, accompanied by a courtship dance (e.g., pivoting body from side to side), and associated with the fluffing of the male’s plumage [41–42]. We monitored the male’s behavior toward a female via video and scored songs as FD songs if they were accompanied by at least two of the above behaviors. The median interval between exposures to females was 5 minutes, allowing for the collection of interleaved renditions of UD songs. Not all birds, however, produced renditions of UD song between female presentations. Therefore, we also recorded UD songs for 30 min before and after the testing session (~2 hrs) to ensure that UD and FD songs were obtained from roughly the same time of day. We also recorded the UD and FD songs of a subset of these birds as older adults.

To analyze age-dependent changes to song, we used a repeated measures design to compare the UD songs that adult Bengalese finches (n = 22) produced as young adults (mean = 6 months of age; range: 4–11 months) and as older adults (mean = 23 months of age; range: 11–55 months)[34]. After young adult recordings, birds were housed either with other males in a group cage (n = 15) or with a single female in a breeding cage (n = 7). The relationship between syllable sequencing and timing was not significantly different between group-housed and breeding males (p>0.60); therefore, data from both groups were combined in the analysis. For each time point and each bird, we analyzed 30 randomly selected songs during the day, except for two birds where only 22 and 25 songs were recorded as older adults. It should be noted that Bengalese finches can live up to 10 years of age in laboratory conditions; therefore, we are not studying senescence and the deterioration of song (e.g., [43]).

Data from these birds were obtained from recordings used in previous analyses of syllable sequencing at branch points and syllable timing within stereotyped sequences [34, 35]. However, context- and age-dependent changes to syllable timing at branch points have not previously been analyzed or reported. The current investigation expands existing analyses of context- and age-dependent changes to song and allows for the systematic examination of the relationship between the control and plasticity of syllable sequencing and timing.

### Song Analysis

Bengalese finch song consists of acoustically distinct elements (‘syllables’) separated by ≥5 ms of silence (e.g., [16, 34, 35, 38]). We manually labeled individual song syllables based on visual inspection of spectrograms following amplitude-based syllable segmentation (e.g., [16, 19, 30, 34–36, 38, 44, 45]).

**Fig 1:**
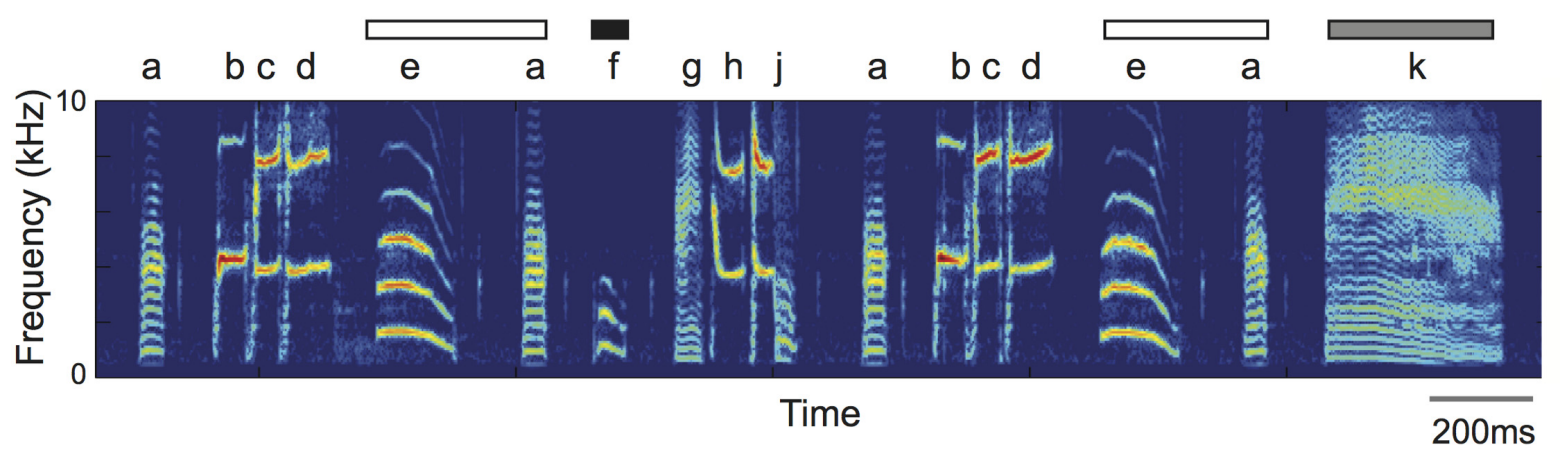
Bengalese finch song. Bengalese finch song consists of distinct acoustic elements (‘syllables’) that are arranged into stereotyped sequences as well as sequences that vary in transitions from rendition to rendition (‘branch points’). Depicted is a spectrogram (frequency on the y-axis, time on the x-axis, color as intensity) of a single rendition of an adult Bengalese finch song, with letters labeling individual syllables in the song. In this example, the sequences ‘bcd’ and ‘ghj’ are stereotyped sequences. The sequence ‘ea’ is a branch point because transitions from ‘ea’ vary across renditions: the bird transitions to ‘f’ 90% of the time and ‘k’ 10% of the time. We analyzed how the probabilities of individual syllable transitions co-varied with the duration of silent gaps between syllable transitions (e.g., gaps between ‘a’ and ‘k’ and between ‘a’ and ‘f’).

Adult Bengalese finch song consists of syllables arranged in stereotyped sequences as well as sequences in which syllable transitions vary from rendition to rendition (‘branch points’; Fig 1)[30, 45–46]. Stereotyped and branch point sequences were identified during manual labeling and confirmed using bigram plots (e.g., [19, 38–39, 47]). Stereotyped sequences are sequences of syllables in which transitions between pairs of syllables occurred >95%.

Branch points are nodes in song in which syllable transitions varied across renditions. Typically, there are 2–5 transitions at individual branch points, and variability in sequencing at branch points has been determined not to be biological noise, but a controlled feature of song [16, 19, 30, 36, 45]. We computed the probability of individual transitions at branch points, excluding song terminations from this calculation. We paid close attention to long-range statistics (i.e., history dependence) in Bengalese finch song (e.g., [19, 45, 48–49]) and analyzed sequencing at branch points based on these sequence dependencies. For example, if a branch point sequence ‘cd’ was preceded either by an ‘a’ or a ‘b’, we analyzed ‘acd’ and ‘bcd’ sequences separately if transition probabilities were significantly different across sequence contexts (Likelihood ratio tests, α = 0.05); otherwise, we pooled across sequence contexts for the analysis. Branch points were independently identified for each dataset (e.g., UD and FD song, young and older adults) and analyzed only if they occurred at least 10 times under each condition. Age- and context-dependent changes were analyzed only for sequences considered to be branch points under at least one of the conditions. Variation in the sequencing of syllables that are repeated a variable number of times across renditions were not analyzed.

Changes to song tempo are driven primarily by changes to the duration of the silent interval between syllables [14, 33–35]. Therefore, we calculated the duration of silent intervals between syllables at branch points and analyzed how variation in gap durations related to variation in syllable sequencing. Following amplitude-based segmentation, we measured gap durations as the interval from the offset of the first syllable in the transition to the onset of the subsequent syllable. We then computed the median gap duration for each transition at branch points. Because small sample sizes affect the reliability of the estimate of central tendencies, we only analyzed median gap durations for transitions with ≥5 instances.

Context- and age-dependent changes to gap durations were examined using slightly different methods due to variation in data collection. Because interleaved renditions of UD and FD song were collected on the same day and under the same recording conditions, we used the same amplitude threshold on raw sound envelopes to compute gap durations for UD and FD songs. On the other hand, because young and older adult recordings were separated by years and because recording conditions could have been different between recordings, we analyzed normalized waveforms across recordings as per previous studies [34, 35, 43]. To normalize the data, we extracted the waveform of each rendition of a sequence transition of a branch point (e.g., each rendition of the transitions ‘af’ and ‘ak’ for the bird depicted in Fig 1) then resampled (1 kHz), smoothed (5 ms square window), and rectified the waveform. Thereafter, we computed the median envelope across all examples of the transition, then normalized this sound envelope such that the branch point syllable (i.e., syllable transitioned from) had the same peak amplitude across recordings. After this normalization, the amplitude traces were comparable across ages, and we used a common threshold on these normalized traces to compute and compare gap durations.

Because amplitude can affect threshold-based measures of gap durations, we also analyzed how the amplitude of transition syllables (i.e., syllable transitioned to) co-varied with gap durations and transition probabilities. For the analysis of the relationships between transition probability and syllable amplitudes, we computed the root mean square (RMS) of the baseline (silence)-subtracted waveform for each transition syllable at all branch points in which gap durations were measured. Specifically, we computed the RMS from the onset to offset of each transition syllable as determined by amplitude thresholds used to compute gap durations and calculated the median RMS for each syllable. To normalize values across the different song recording platforms, we divided the median RMS values of each transition syllable by the maximum value of each recording platform. For the analysis of age-dependent changes to song, we calculated the RMS of transition syllables from the normalized, median waveforms used to compare gap durations across age (see above). Because these waveforms were normalized such that the peak amplitude of the branch point syllable (i.e., syllable transitioned from) was equal across ages, these values represent the amplitudes of transition syllables relative to the branch point syllable.

### Statistical analysis

The distribution of median gap durations and syllable amplitudes were generally not normally distributed (Shapiro-Wilks, p<0.05) and were log_10_-transformed to improve normality. In most cases, the log_10_ transformation normalized the distribution. However, in some cases the transformation reduced the skewness and kurtosis of the distribution but the distribution remained significantly different from normal. We continue to analyze the log_10_-transformed values in these instances for consistency across analyses and because the transformation generally improved the normality of the distribution.

To analyze differences in gap durations between stereotyped and branch point sequences, we used a mixed effects model with transition type (stereotyped vs. branch point) as the independent variable and log_10_ gap durations as the dependent variable. Because multiple branch points and stereotyped sequences were measured within a bird and because multiple gaps were measured within each branch point and stereotyped sequence, we also included bird ID and sequence ID nested within bird ID as random factors in the model. For all mixed effects analyses we used a restricted maximum likelihood method (REML) with unbounded variance components.

We used a model with the same random variables to analyze the relationships between transitions probabilities, inter-syllable gap durations, and syllable amplitude at branch points (see Results).

To analyze the relationship between age- and context-dependent changes to transition probabilities and gap durations, we first computed the difference in transition probabilities as well as the difference in log_10_ gap durations between relevant datasets (e.g., young vs. older adult, UD vs FD song). As in the previous analysis, only transitions with ≥5 instances in both datasets were included in the analysis of age- and context-dependent changes. We then used mixed effects models to analyze the degree to which changes in gap durations at branch point transitions were linearly related to changes in the probability of those transitions. For these analyses, the change in gap durations (e.g., log_10_ gap duration for older birds minus log_10_ gap durations for young birds) was the dependent variable and the change in the probability of that transition (e.g., transition probability for older birds minus transition probability for young birds) was the independent variable. As with the previous model, we also included bird ID and sequence ID nested within bird ID as random factors in the model. Because young adult Bengalese finches tend to prune branch point transitions over time [34], there are fewer transitions in the analysis of age-dependent changes than in the analysis of young adult song. A similar model was used to analyze age-dependent changes to syllable amplitude (e.g., dependent variable: log_10_ syllable amplitude for older birds minus log_10_ amplitude for young birds).

To complement the analysis of age-dependent changes to gap durations, we also analyzed the percent change in median gap durations for transitions that changed in prevalence in different ways. We first analyzed the degree to which the probabilities of individual transitions changed over time using a χ^2^ test, and then categorized branch point transitions as transitions that significantly increased in prevalence, significantly decreased in prevalence, or remained the same (α = 0.05). We also included data from stereotyped sequences within each bird (data from [34]) with the hypothesis that the magnitude of change in gap durations would be comparable between stereotyped transitions (which remained stereotyped over time) and branch point transitions that did not change in transition probability over time (see Results). For this model, the independent variable was transition type (branch point transitions that significantly increased in prevalence, branch point transitions that significantly decreased in prevalence, branch point transitions that remained the same in transition probability, and stereotyped transitions) and the dependent variable was the percent change in median gap durations. Bird ID and sequence ID nested within bird ID were included as random factors.

Analyses were done using JMP 11.0 (SAS Institute, Cary, NC) or Matlab, and α = 0.05 for all tests.

## Results

### Relationship between transition probability and gap durations

**Fig 2:**
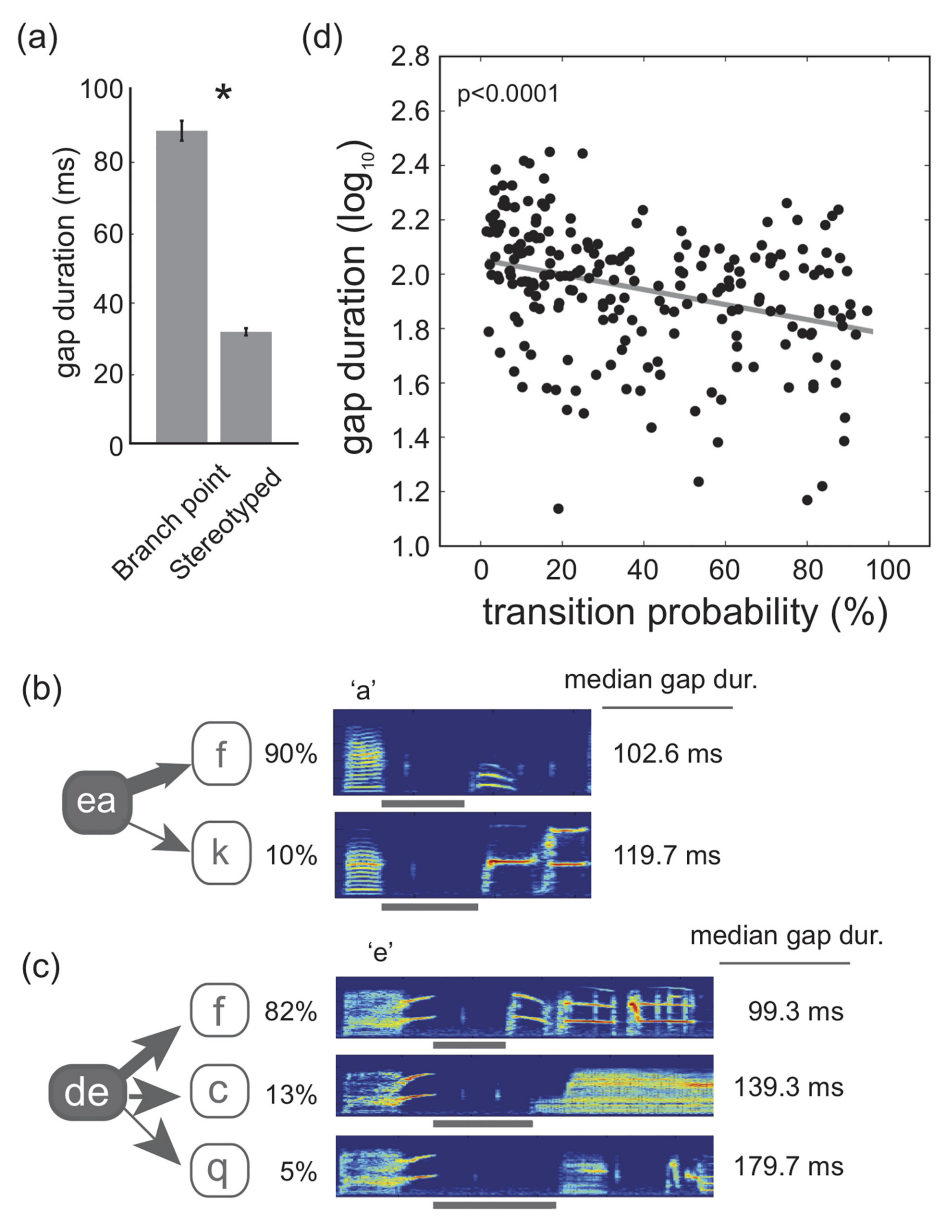
Inverse relationship between transition probability and gap durations. (a). Gap durations were significantly shorter for transitions within stereotyped sequences (transition probabilities >95%) than for transitions within branch points (transition probabilities <95%). Analyses were conducted on log_10_-transformed data but presented here are back-transformed means. (b). An example of variation in gap durations as a function of transition probability within a branch point. The bird transitioned from ‘ea’ to ‘k’ on 10% of renditions and to ‘f’ on 90% of renditions (same branch point depicted in Fig 1). The duration of the gap between ‘a’ and ‘k’ (the less prevalent transition) was longer than the gap between ‘a’ and ‘f’ (the more prevalent transition). Thickness of arrows relates to the prevalence of that transition. (c). Another example of variation in gap durations as a function of transition probability. The bird transitioned from ‘de to ‘f’ on 82% of renditions, to ‘c’ on 13% of renditions, and to ‘q’ on 5% of renditions. The duration of the gap between ‘e’ and ‘f’ (the most prevalent transition) was shortest, while the gap was longest between ‘e’ and ‘q’ (the least prevalent transition). Thickness of arrows relates to the prevalence of that transition. (d). There was a significant negative relationship between transition probability and log_10_ gap duration (F_1,144.6_ = 29.1, p<0.0001; slope = -0.240±0.044).

Bengalese finch song consists of stereotyped sequences of syllables as well as sequences with variable transitions (branch points). Stereotyped sequences consist of transitions in which transitions probabilities are >95%, whereas branch points are defined as sequences in which transition probabilities are <95%. To analyze the relationship between transition probability and gap durations at a broad level, we analyzed the undirected songs of young adult Bengalese finches (n = 22 birds; 4–11 months of age) and compared gap durations within branch points (n = 218 gaps from 86 branch points) to gap durations within stereotyped sequences (n = 107 gaps from 42 stereotyped sequences)[34]. Overall, gap durations within stereotyped sequences wherein transition probabilities were >95% were significantly shorter than gap durations at branch points wherein transition probabilities were <95% (F_1,98.3_ = 135.0, p<0.0001; Fig 2A). This indicates an inverse relationship between transition probabilities and gap durations at a broad level.

To investigate the relationship between transition probabilities and gap durations in greater detail, we examined the degree to which transition probabilities of individual transitions within branch points were related to the duration of the gap within each transition. To this end, we analyzed the timing and sequencing of syllables within spontaneous songs produced in isolation by young adult Bengalese finches (218 transitions from 86 branch points in 22 males). Median gap durations ranged from 13.8 to 489.1 ms, with an average of 111.7 ms across all birds. In the example provided in Fig 2B, there was an association between transition probabilities within the branch point and inter-syllable gap durations. In this example, the bird transitioned to the syllables ‘k’ and ‘f’, respectively, on 10% and 90% of the time following the branch point ‘ea’. The median interval between ‘a’ and the more prevalently produced ‘f’ transition was 102.6 ms, whereas the median interval between ‘a’ and the less prevalently produced ‘k’ transition was 119.7 ms. In another branch point with three transitions, there was similarly an inverse relationship between transition probabilities and gap durations (Fig 2C). This relationship was common across branch points, and as such, there was an overall significant inverse relationship between log_10_ gap durations and transition probabilities (F_1,144.6_ = 29.1, p<0.0001; slope = -0.240±0.044; Fig 2D; see also S1 Fig). Transitions with higher transition probabilities were associated with short gaps, whereas transitions with lower transition probabilities were associated with longer gaps. This finding is consistent with previous studies and suggests that syllable sequencing and timing could be functionally coupled [32].

Gaps were computed based on amplitude-based segmentation. As such, variation in amplitude could contribute to variation in gap durations. We investigated the contribution of variation in amplitude to the preceding results by analyzing the relationship between transition probability and the amplitude of transition syllables (i.e., syllables after the gap; log_10_). There was a significant and positive relationship between transition probabilities within branch points and the amplitude of transition syllables (F_1,148.9_ = 14.6, p = 0.0002): transition syllables that were less frequently produced were lower in amplitude (S2 Fig). Similarly, there was a significant inverse relationship between syllable amplitude and log_10_ gap durations (F_1,186.6_ = 39.4, p<0.0001; S2 Fig). These analyses suggest that variation in amplitude could mediate the relationship between variation in transition probabilities and gap durations. To assess the relationship between transition probabilities and gap durations *independent* of syllable amplitude, we first computed the residuals from the analysis of the relationship between syllable amplitude and gap duration; these residuals represent variation in gap durations that is not accounted for by variation in amplitude. Thereafter, we analyzed the relationship between transition probability and these residuals and found a significant and inverse relationship between transition probability and residual variation in log_10_ gap durations (F_1,147.7_ = 16.4, p<0.0001; S2 Fig). This indicates that there is a significant relationship between transition probability and gap durations independent of variation in amplitude.

### Relationship between age-dependent changes to syllable timing and sequencing

Young adult Bengalese finches have relatively stable songs compared to juveniles, but the temporal structure of adult song continues to change over time [34]. For example, the duration of stereotyped sequences of syllables becomes significantly shorter with age in Bengalese finches, and this change is due primarily to a shortening of inter-syllable gaps. However, the degree to which gap durations at branch points change with age remains unknown. To this end, we compared gap durations of branch point transitions that individual birds produced as young adults and as older adults (n = 125 transitions from 76 branch points in 22 birds). Similar to the effect observed within stereotyped sequences [34], gaps between transitions at branch points became significantly shorter with age (F_1,122.4_ = 17.6, p<0.0001).

**Fig 3:**
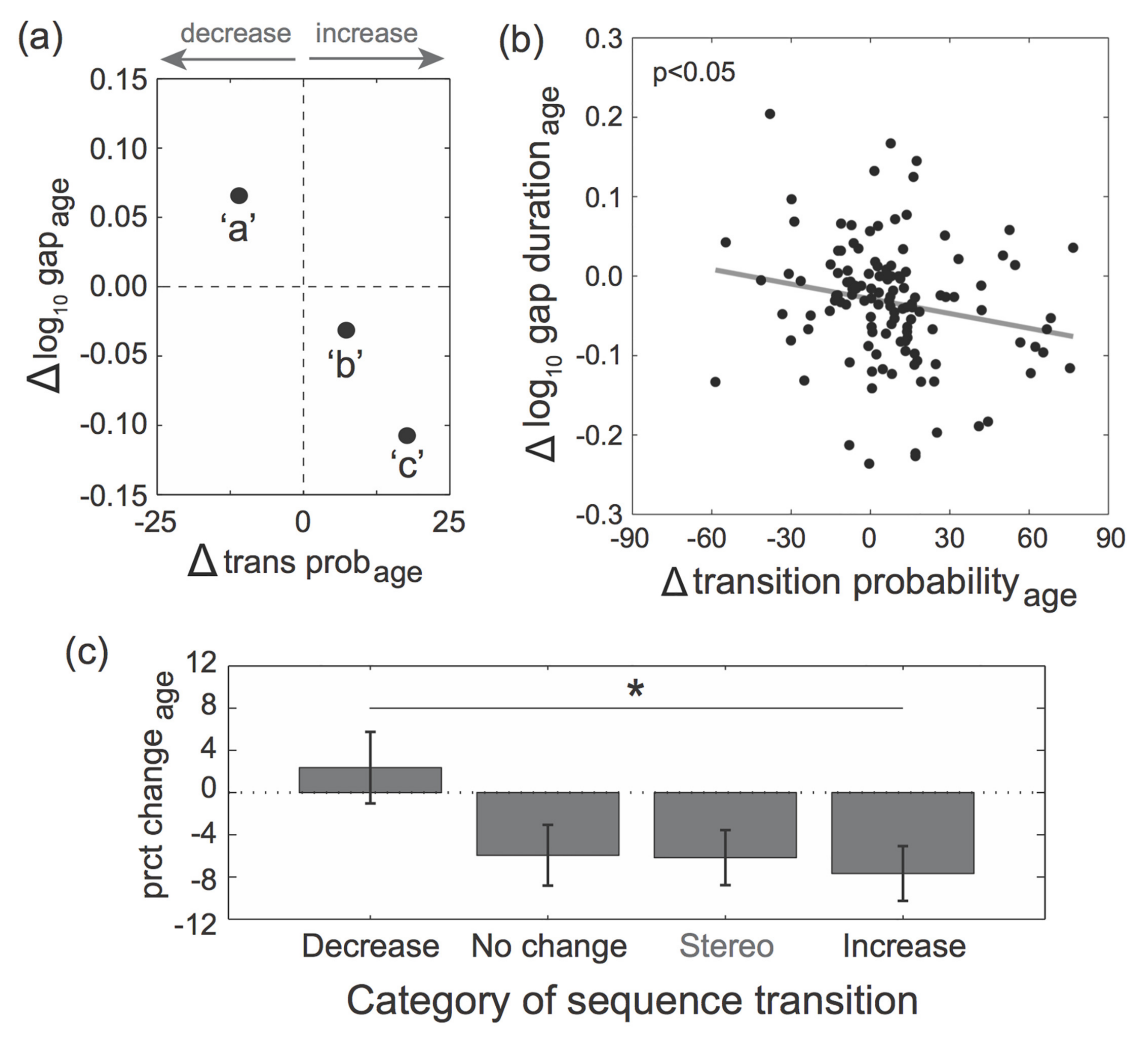
Relationship between age-dependent changes to transitions probabilities and gap durations within branch points. (a). An example of age-dependent changes to gap durations as a function of age-dependent changes to transition probabilities. Gap durations decreased for the two transitions that increased in prevalence whereas the duration of the gap for the transition that became less prevalent over time increased. (b). There was a significant negative relationship between age-dependent changes to transition probabilities and log_10_ gap durations (F_1,108.4_ = 5.3, p = 0.0230; slope = -0.063±0.027). (c). The magnitude of change (percent change) in gap durations varied depending on whether the prevalence of a transition within a branch point significantly decreased, did not significantly change, or significantly increased (Likelihood ratio test) over time. Also included in the analysis was the percent change in gap durations for transitions within stereotyped sequences (data from [34]). The magnitude of change in gap durations was significantly different between transitions that significantly decreased and transitions that significantly increased in transition probability over time (Tukey’s HSD, p<0.05) and nearly identical between stereotyped transitions and branch point transitions that did not change in probability over time.

We next analyzed the relationship between age-dependent changes to syllable sequencing (difference in transition probabilities between young and older adults) and to gap durations. Transition probabilities at branch points change significantly over time [34], but it is unclear whether changes in the likelihood of a particular transition are systematically related to changes in the duration of the gap within the transition. An example of age-dependent changes to syllable sequencing and gap durations is depicted in Fig 3A. In this example, the transition probabilities of the three transitions of the branch point changed by +18%, +7%, and -11% as the bird aged from 8 months to 26 months of age. (The bird initially sang three other transitions that were produced on 14% of renditions, in total, and each of these transitions was pruned over time.) Correspondingly, the log_10_ gap durations of these transitions changed by -0.11, -0.03, and +0.07, respectively. As such, gap durations decreased for the two transitions that increased in probability over time whereas the gap duration increased for the transition that became less prevalent over time. Such trends in the changes to syllable sequencing and gap durations were common across branch points. As such, there was a significant negative relationship between the change in transition probabilities and the change (difference) in log_10_ gap durations (Fig 3B; F_1,108.4_ = 5.3, p = 0.0230; slope = -0.063±0.027). There was similarly a significant negative relationship between age-dependent changes to transition probabilities and percent changes to gap durations (F_1,110_ = 5.7, p = 0.0184; slope = -14.4±6.0). Together with the finding that gap durations significantly decreased over time, this indicates that gap durations decreased more for transitions that increased in probability over time than for transitions that decreased in probability over time.

As indicated above, variation in gap durations could be mediated, in part, by variation in syllable amplitude. However, changes to transition probabilities across age were not systematically related to age-dependent changes to the relative amplitude of transition syllables (see Methods; F_1,97.3_ = 0.1, p = 0.7664). Similarly, variation in age-dependent changes to gap durations were not significantly related to variation in age-dependent changes to relative amplitude (F_1,106.6_ = 0.7, p = 0.4140). This suggests that the age-dependent variation in amplitude did not significantly contribute to the relationship between age-dependent changes to transition probabilities and gap durations.

To further investigate this relationship, we compared the magnitudes of change in gap durations (percent change) among sequence transitions within branch points and stereotyped sequences. We categorized sequence transitions within a branch point as transitions that significantly increased, significantly decreased, or did not change in prevalence over time (Likelihood ratio test, α = 0.05). We then compared the magnitude of change in gap durations among these types of transitions as well as the magnitude of change in gap durations for previously identified stereotyped sequences (data from [34]). Because gaps are significantly longer within branch points than within stereotyped sequences, we analyzed the percent change in gap durations over time so as to normalize for baseline differences in durations across categories. We hypothesized that branch point transitions that significantly increased in prevalence over time would demonstrate the largest decrease in gap durations whereas branch point transitions that significantly decreased in prevalence over time would demonstrate the smallest decrease in gap durations. Furthermore, because sequencing within stereotyped sequences does not change over time [34], we hypothesized that the magnitude of change in gap durations for branch point transitions that did not significantly change in prevalence over time would be indistinguishable from the magnitude of change in gap durations within stereotyped sequences. Consistent with these predictions, the magnitude of change to gap durations significantly varied across these types of transitions (F_3,114.2_ = 3.0, p = 0.0353; Fig 3C). In particular, transitions that significantly increased in transition probability over time demonstrated the largest decrease in gap durations, and this change in gap durations was significantly different than the change in gap durations for transitions that significantly decreased in transition probability over time (Tukey’s HSD, p = 0.0204). Importantly, the magnitude of change of gap durations for branch point transitions that did not change over time (-6.0±2.9%) was nearly identical to the magnitude of change for stereotyped transitions (-6.2±2.6%; p>0.90). These analyses further bolster the relationship between plasticity in syllable sequencing and in timing.

### Relationship between context-dependent changes to syllable timing and sequencing

It has previously been reported that the duration of stereotyped sequences of syllables is significantly shorter when male Bengalese finches produce courtship songs to females than when they produce spontaneous song in isolation [16, 19, 35]. However, the degree to which syllable timing within branch points changes across social contexts remains unknown. To this end, we compared gap durations at branch points when young adult Bengalese finches produced female-directed (FD) songs or undirected (UD) songs (n = 74 gaps from 27 branch points in 14 birds). Similar to the effect observed for stereotyped sequences [35], gap durations at branch points were significantly shorter when birds produced FD song than UD song (F_1,73_ = 4.1, p = 0.0453), supporting the notion that birds speed up their songs when courting females.

**Fig 4:**
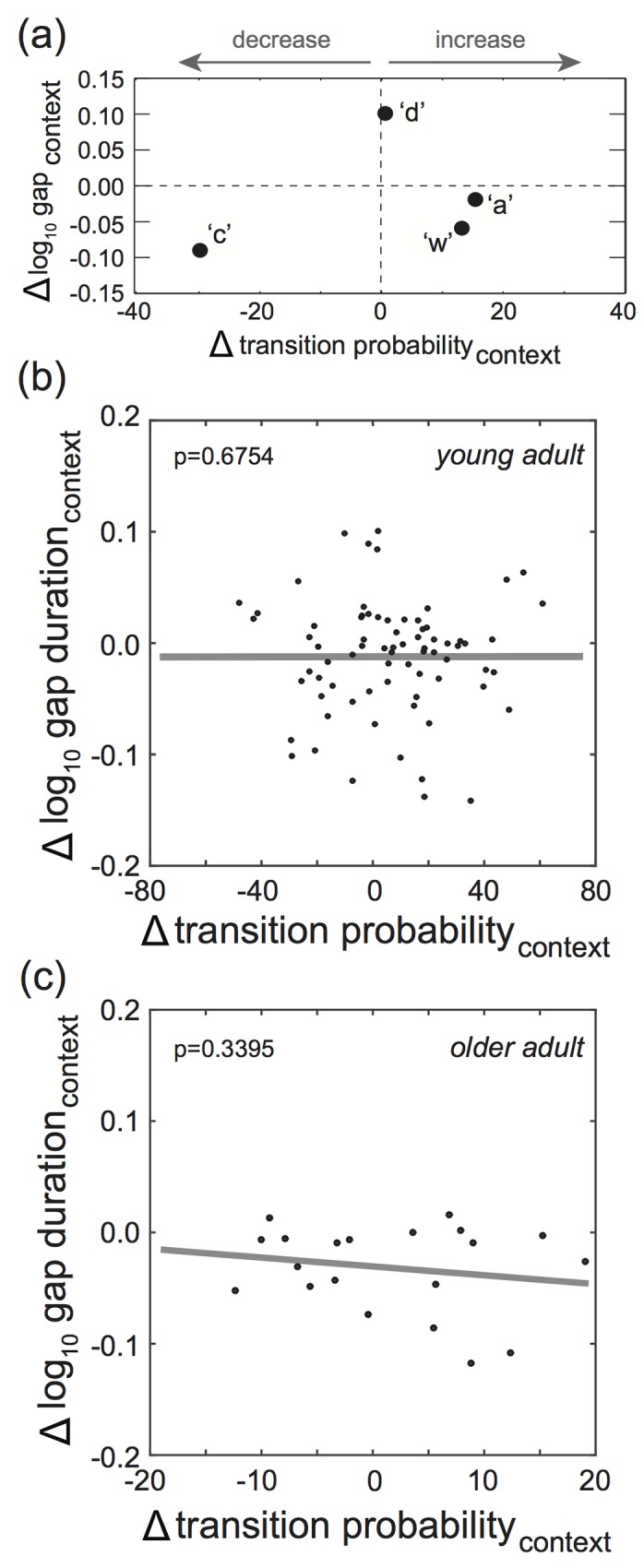
Relationship between social context-dependent changes (from undirected (UD) to female-directed (FD) song) to transitions probabilities and gap durations. (a). An example of variation in context-dependent changes to gap durations as a function of context-dependent changes to transition probabilities. Relative to when the young adult bird produced UD song, the transition probability to ‘c’ decreased whereas transitions to ‘a’, ‘d’, and ‘w’ increased in prevalence by varying degrees when the bird produced FD song. Gap durations decreased for all transitions except to ‘d’, indicating little correspondence between changes to transition probabilities and gap durations in this example. (b). Overall, there was no significant relationship between context-dependent changes to transition probabilities and to log_10_ gap durations for young adult Bengalese finches (F_1,53.2_ = 0.0, p = 0.9925). (c). Similar to young adults, there was no significant relationship between context-dependent changes to transition probabilities and to log_10_ gap durations for older Bengalese finches (F_1,7.9_ = 1.0, p = 0.3395). Data on transition probabilities at branch points from [35].

We next analyzed the relationship between context-dependent changes to transition probabilities and gap durations. Within both UD and FD song, there was a significant inverse relationship between log_10_ gap durations and transition probabilities (S1 Fig). However, there was little evidence that context-dependent changes to transition probabilities were related to context-dependent changes to gap durations. In the example provided in Fig 4A, the bird produced four transitions after the branch point that changed in transition probability by +15%, +13%, +2%, and -30% from UD to FD song. Correspondingly, log_10_ gap durations changed by-0.06, -0.02, +0.10, and -0.09, respectively, for these transitions. As such, gap durations decreased for transitions that decreased or increased in prevalence and increased for the transition that exhibited minimal change in transition probability. Therefore, there was little relationship between changes to transition probabilities and gap durations in this example. There was little consistency across branch points in how context-dependent changes to gap durations related to changes to transition probabilities. Consequently, across all branch point transitions, there was no significant relationship between context-dependent changes to transition probabilities and to log_10_ gap durations (F_1,53.2_ = 0.0, p = 0.9925; slope = 0.000+0.025; Fig 4C). This lack of significant relationship was also observed when percent changes to gap durations were analyzed (F_1,53.8_ = 0.0, p = 0.9949; slope = 0.0±5.6) and indicate that gap durations at branch points acutely decreased from UD to FD song in a manner that was independent of changes to syllable sequencing.

We similarly analyzed the relationship between context-dependent changes to gap durations and syllable sequencing at branch points for a subset of Bengalese finches when they were older adults (n = 20 transitions from 6 birds). As observed for young adults, there was no significant relationship between the magnitude of change to transition probabilities and to log_10_ gap durations (F_1,7.9_ = 1.0, p = 0.3395; slope = -0.054±0.053; Fig 4C).

## Discussion

Coordinating the sequencing and timing of motor gestures is important for the display of behavior [1–5, 8, 11]. Here we investigated the relationship between the sequencing and timing of acoustic elements (‘syllables’) within the songs of adult Bengalese finches. We focused our analysis on branch points, nodes in song in which syllable transitions varied from rendition to rendition, and analyzed the degree to which the probabilities of sequence transitions were related to the durations of the silent gap between syllables within the transitions. We found that the probabilities of transitions between syllables at branch points were systematically and inversely related to the duration of silent gaps between syllables in the transition. In general, gaps were shorter for transitions that were more frequently produced, a finding that is consistent with a previous study (Fig 2; S1 Fig)[32]. Furthermore, we extend these findings by documenting that age- but not context-dependent changes to syllable sequencing and timing follow this inverse relationship (Figs 3 and 4). This suggests that the relationship between syllable sequencing and timing could shape the nature of motor plasticity but also that the moment-by-moment control of syllable sequencing could be independent of the moment-by-moment control of syllable timing.

The inverse relationship between transition probabilities and gap durations at branch points is consistent with models of motor sequence production, including models of song control. General models of motor control propose that motor sequencing is regulated by functional connections between populations of neurons that control individual motor gestures (e.g., [4, 11, 50–52]). Furthermore, these models propose that the strength of functional connections between such populations reflect the probability of producing such sequences as well as the speed at which these motor sequences can be performed: stronger functional connections represent more frequently produced motor sequences that can be produced at a faster speed.

Models of song control share many features with general models of motor control. Song is regulated by neural activity in the song system, a collection of circuitry including forebrain, thalamic, and hindbrain areas, and a number of models of song control focus on the forebrain nucleus HVC (used as proper name), an area that is functionally similar to the premotor cortex in mammals [12,13,21,23,27,50]. It has been proposed that populations of neurons in HVC encode individual syllables and that functional connections between such populations regulate the sequencing of vocal motor gestures (e.g., syllables)[13–15, 30, 33, 46, 53–56]. These ideas are based on findings that individual motor projection neurons in HVC are active during a single part of song (e.g., burst during a single syllable), that different HVC projection neurons are active during different parts of song, and that HVC projection neurons are functionally coupled [57–60]. Additionally, manipulations that affect syllable sequencing and timing affect HVC activity, and perturbations of HVC activity drive changes to syllable sequencing and timing [37, 53–56, 61].

To explain more complex and variable forms of syllable sequencing (e.g., within Bengalese finch song), models of sequence generation have proposed that variation in transition probabilities between syllables reflect variation in the functional connectivity between populations of projection neurons that encode individual syllables (e.g., [46, 49]). In particular, it has been proposed that sequence transitions that are more prevalent are associated with stronger functional connections between populations of neurons encoding syllables. These models generally have not explicitly linked mechanisms of syllable sequencing to mechanisms of syllable timing, but given other models of motor control, we hypothesized that the control and plasticity of syllable sequencing could be related to the control and plasticity of syllable timing (e.g., [4, 11]). Our data demonstrating an inverse relationship between transition probabilities and gap durations and between age-dependent plasticity in transition probabilities and gap durations support such models. Indeed, our finding that age-dependent changes to the frequency of individual transitions correlate with age-dependent changes to the duration of gaps within those transitions suggests a singular mechanism could underlie such long-term changes to song (e.g., [33]). Specifically, age-dependent changes to the temporal patterning of song could reflect changes to the functional strengths of neural populations encoding syllables, leading to correlated changes to sequencing and timing.

In contrast to the significant relationship between age-dependent changes to syllable sequencing and timing, we did not find a systematic relationship between acute context-dependent changes to syllable sequencing and timing. Gap durations decreased to the same extent regardless of the direction and magnitude of context-dependent changes to the probability of transitions. This dissociation suggests that mechanisms that underlie acute changes to syllable sequencing could be distinct from mechanisms underlying acute changes to syllable timing. Given the models of sequence generation in HVC, it is not clear how this dissociation occurs, but this finding is consistent with a previous study documenting that administration of amphetamine acutely affects syllable sequencing at branch points without affecting song tempo [36]. Neural activity in song control areas outside of HVC can also contribute to syllable sequencing or timing, and it is possible that social context acts on such circuits to produce these independent changes. For example, social context affects immediate early gene expression, a cellular marker of neural activity, not only in the HVC of Bengalese finches but also in a number of song control nuclei, including RA (robust nucleus of the arcopallium), an area that receives inputs from HVC and that is homologous to the primary motor cortex [62]. Neural activity in RA has been found to influence syllable sequencing (e.g., [54, 63]), and it is possible that context-dependent changes to RA activity affects sequencing in a manner that is independent of syllable timing. Despite our lack of understanding of mechanisms of context-dependent changes to song, our data emphasize that models of song control should not only include mechanisms that link syllable sequencing and timing but also incorporate mechanisms that independently affect sequencing and timing.

In addition to providing insight into mechanisms of vocal motor control and plasticity, our analyses could be informative for understanding the regulation of other complex motor behaviors such as music or language. For example, experience and practice increase a musician’s ability to play musical pieces at a faster tempo [29, 64–66]. Similarly, practice leads to a decrease in the frequency of sequencing errors during the performance of a musical piece (i.e., sequencing becomes more stereotyped) [64–65]. Little is known about the degree to which practice-dependent increases in the tempo of performance correlate with practice-related decreases in sequencing errors, but our data suggest a possible relationship between the two types of practice-related changes. Similarly, increases in language fluency are characterized by increases in the speed at which individuals can accurately produce language as well as decreases in the frequency of syntactic errors (e.g., [67–71]), and it is also possible that there is a systematic relationship between the magnitudes of such changes.

## Supporting Information

S1 Fig Inverse relationship between transition probabilities and gap durations (log_10_) at branch points. (a). Undirected songs of young adults (n = 218 transitions from 22 birds; F_1,144.6_ = 29.1, p<0.0001; slope = -0.240±0.044; same as Fig 2D). (b). Undirected songs of older adults (n = 144 transitions from 22 birds; F_1,98.3_ = 36.1, p<0.0001; slope = -0.372±0.062). (c,d) Undirected (c; n = 107 transitions from 14 birds; F_1,64.8_ = 7.1, p = 0.0097; slope = -0.150±0.056) and female-directed (d; n = 73 transitions from 14 birds; F_1,43.4_ = 14.1, p = 0.0005; slope = -0.253±0.067) songs of young adults collected an interleaved manner. For all plots, gap durations were computed only for transitions in which n≥5. Sample sizes differ between young (a) and older (b) adults because sequencing at branch points become more stereotyped over time and because some transitions are pruned over time [34]. Similarly, sample sizes differ between undirected and female-directed songs, in part, because of changes to the stereotypy of sequence transitions [35]. There are more data points in these analyses than for the analyses of age- and context-dependent change because the latter analyses require that sequence transition have ≥5 instances under *each* condition.
(PDF)

S2 Fig Relationships between transition probabilities, gap durations, and syllable amplitude in the undirected songs of young adults. (a). Transition probabilities are significantly and positively related to the amplitude of transition syllables (i.e., syllables transitioned to; log_10_). (b). Gap durations are inversely related to the amplitude of transition syllables. (c). There remained a significant inverse relationship between transition probabilities and gap durations after taking into account the relationship between gap durations (log_10_) and syllable amplitude [i.e., residuals from (b)].
(PDF)
