# Supplementary material for: Relationship between the Sequencing and Timing of Vocal Motor Elements in Birdsong
Source: PLoS One. 2015 Dec 9;10(12):e0143203. doi: 10.1371/journal.pone.0143203 (PMC4674110; doi:10.1371/journal.pone.0143203)
Supplement: S1 Fig — (a). Undirected songs of young adults (n = 218 transitions from 22 birds; F1,144.6 = 29.1, p<0.0001; slope = -0.240±0.044; same as Fig 2D). (b). Undirected songs of older adults (n = 144 transitions from 22 birds; F1,98.3 = 36.1, p<0.0001; slope = -0.372±0.062). (c,d) Undirected (c; n = 107 transitions from 14 birds; F1,64.8 = 7.1, p = 0.0097; slope = -0.150±0.056) and female-directed (d; n = 73 transitions from 14 birds; F1,43.4 = 14.1, p = 0.0005; slope = -0.253±0.067) songs of young adults collected an interleaved manner. For all plots, gap durations were computed only for transitions in which n≥5. Sample sizes differ between young (a) and older (b) adults because sequencing at branch points become more stereotyped over time and because some transitions are pruned over time [34]. Similarly, sample sizes differ between undirected and female-directed songs, in part, because of changes to the stereotypy of sequence transitions [35]. There are more data points in these analyses than for the analyses of age- and context-dependent change because the latter analyses require that sequence transition have ≥5 instances under each condition. (PDF) [file pone.0143203.s001.pdf]

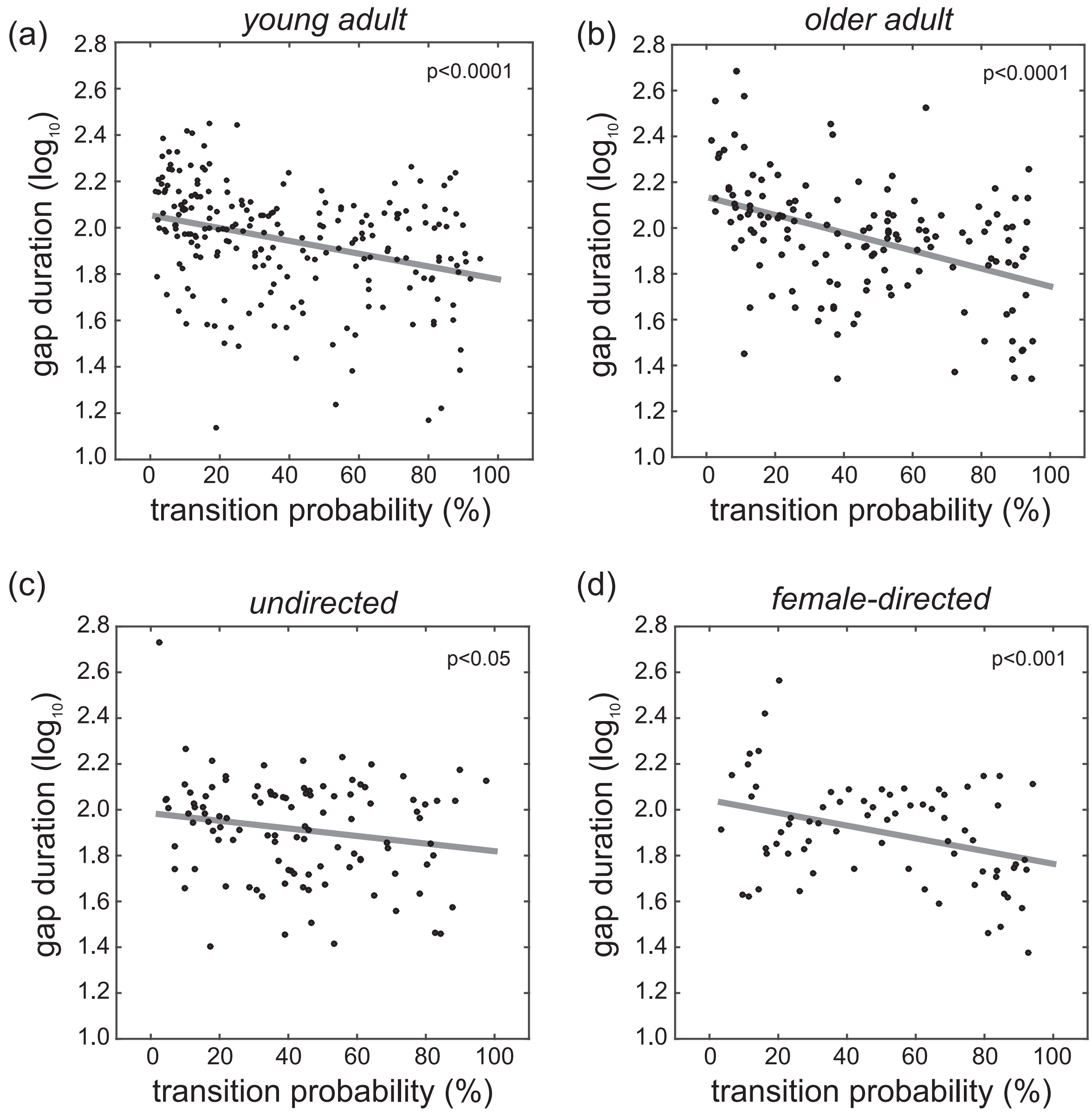

S1 Fig. Inverse relationship between transition probabilities and gap durations ($\log_{10}$) at branch points. (a). Undirected songs of young adults (n=218 transitions from 22 birds; $F_{1,144.6}$=29.1, p<0.0001; slope=-0.240±0.044; same as Figure 2d). (b). Undirected songs of older adults (n=144 transitions from 22 birds; $F_{1,98.3}$ =36.1, p<0.0001; slope=-0.372±0.062). (c,d) Undirected (c; n=107 transitions from 14 birds; $F_{1,64.8}$=7.1, p=0.0097; slope=-0.150±0.056) and female-directed (d; n=73 transitions from 14 birds; $F_{1,43.4}$=14.1, p=0.0005; slope=-0.253±0.067) songs of young adults collected an interleaved manner. For all plots, gap durations were computed only for transitions in which n≥5. Sample sizes differ between young (a) and older (b) adults because sequencing at branch points become more stereotyped over time and because some transitions are pruned over time [34]. Similarly, sample sizes differ between undirected and female-directed songs, in part, because of changes to the stereotypy of sequence transitions [35]. There are more data points in these analyses than for the analyses of age- and context-dependent change because the latter analyses require that sequence transition have ≥5 instances under *each* condition.
