# Supplementary material for: Relationship between the Sequencing and Timing of Vocal Motor Elements in Birdsong
Source: PLoS One. 2015 Dec 9;10(12):e0143203. doi: 10.1371/journal.pone.0143203 (PMC4674110; doi:10.1371/journal.pone.0143203)
Supplement: S2 Fig — (a). Transition probabilities are significantly and positively related to the amplitude of transition syllables (i.e., syllables transitioned to; log10). (b). Gap durations are inversely related to the amplitude of transition syllables. (c). There remained a significant inverse relationship between transition probabilities and gap durations after taking into account the relationship between gap durations (log10) and syllable amplitude [i.e., residuals from (b)]. (PDF) [file pone.0143203.s002.pdf]

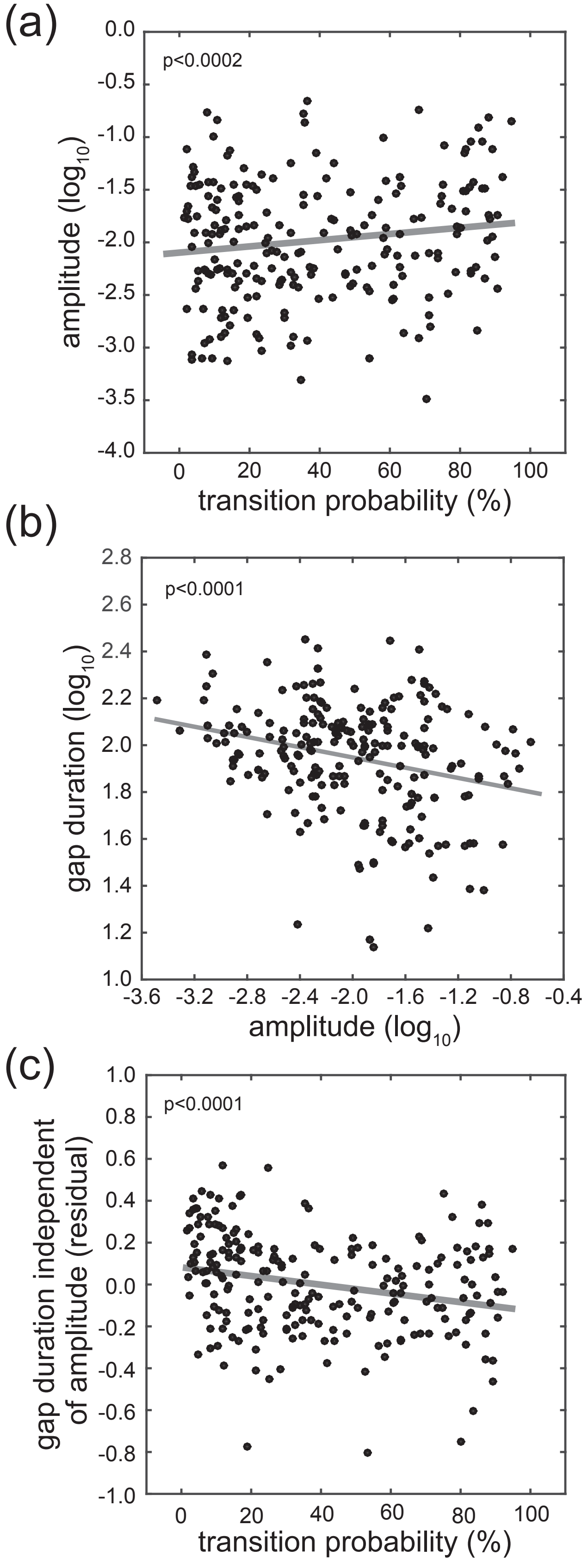

S2 Fig. Relationships between transition probabilities, gap durations, and syllable amplitude in the undirected songs of young adults. (a). Transition probabilities are significantly and positively related to the amplitude of transition syllables (i.e., syllables transitioned to; log_{10}). (b). Gap durations are inversely related to the amplitude of transition syllables. (c). There remained a significant inverse relationship between transition probabilities and gap durations after taking into account the relationship between gap durations (log_{10}) and syllable amplitude [i.e., residuals from (b)].
